# Microbial Infections and Antimicrobial Resistance in Nepal: Current Trends and Recommendations

**DOI:** 10.2174/1874285801812010230

**Published:** 2018-07-31

**Authors:** Ram H. Dahal, Dhiraj K. Chaudhary

**Affiliations:** 1 Department of Microbiology, Tri-Chandra Multiple Campus, Tribhuvan University, Katmhandu, Nepal; 2 Department of Soil Science, Prithu Technical College, Institute of Agriculture and Animal Science, Tribhuvan University, Lamahi, Dang, Nepal

**Keywords:** Antimicrobial resistance, Microbial infection, Antibiotic susceptibility, MRSA, MDR, Nepal

## Abstract

Antimicrobial resistance is a life threatening challenges to the world. Most of the well-known antibiotics are currently ineffective to several microbial diseases. Ampicillin, metronidazole, amoxicillin, cotrimoxazole, chloramphenicol, ciprofloxacin, nalidixic acid, gentamicin, and ceftazidime are common antibiotics whose resistance pattern has been elevated in recent years. The rise and dissemination of resistant bacteria has contributed in increasing cases of antimicrobial resistance. Multi-drug Resistant (MDR) organism such as *Staphylococcus aureus, Pseudomionas aeruginosa, Escherchia coli*, and *Mycobacterium tuberculosis* are principal problems for public health and stakeholders. Globally, issues of antimicrobial resistance are major concern. In the context of Nepal, insufficient surveillance system, lack of appropriate policy, and poor publications regarding the use of antibiotics and its resistance pattern has misled to depict exact scenario of antimicrobial resistance. This mini-review presents current trends of antibiotic use and its resistance pattern in Nepal. In addition, global progression of antibiotic discovery and its resistance has been covered as well. Furthermore, use of antibiotics and possible ways on improvement of effectiveness have been discussed.

## 1 INTRODUCTION

Antimicrobial agents also called antibiotics are the crucial drugs obtained from microorganisms to prevent and treat bacterial infections. The role of antibiotics came into action when Alexander Fleming discovered the penicillin in 1928 [1]. Most of the (about 75%) antibiotics that are currently in clinical use are obtained from actinobacteria isolated either from soil or water [2-4]. To date, continuous uses of antibiotics have created ineffectiveness to antibiotics, leading global rise in drug-resistant bacteria [5]. In recent years, several microbial infectious diseases are no longer responding to commonly used antimicrobial drugs which have elevated multi-drug resistance. The rise and spread of resistant bacteria is a major threat to public health and a unique challenge to both science and medicine [6]. Multi-drug Resistant (MDR) organisms (*Enterococcus* spp., *Klebsiella* spp., *Enterobacter* spp., *Staphylococcus aureus*, *Pseudomonas aeruginosa*, *Acinetobacter baumannii*, *Propionibacterium acnes*, *Staphylococcus epidermidis*, *Escherichia coli*, and *Mycobacterium tuberculosis*) are considered as clinical threat to human and animals [7-12]. The Center for Disease Control and Prevention (CDC) assessed antimicrobial-resistant microbial infections according to various aspects: clinical impact, economic impact, incidence, 10-year projection of incidence, transmissibility, availability of effective antibiotics, and barriers to prevention [13, 14].

Antimicrobial resistance occurs when pathogenic bacteria degrade antibacterial drugs, alter bacterial proteins, and modify membrane permeability to antibiotics [15]. Taking antibiotics without doctor’s prescription as well as medicating antibiotics unnecessarily for treatment of normal viral illness such as common cold, is a good example for increment of antimicrobial resistance [16, 17]. The CDC estimates that antibiotic resistance is responsible for more than two million infections and 23,000 deaths each year in the United States [18]. The therapeutic consumption of antibiotics is increasing continuously and the demands of antibiotics continue to rise exponentially. In a decade of 2000-2010, the total global antibiotic consumption was raised by 30% [19].

Similar as global issue, the antimicrobial-resistance is also a serious complication in Nepal. However, there are no sufficient surveillance system for tracking current antibiotic use and its resistant pattern in Nepal. In addition, few research and published literatures are not abundant to elucidate current scenario. It is truly difficult to report exact trends of antibiotic use and its resistance in Nepal. Therefore, this review accentuates the antibiotic discovery and resistance, the current trends of antibiotic use, its resistance, and extending antibiotic effectiveness in the context of Nepal.

## 2 ANTIMICROBIAL RESISTANCE OF VARIOUS MICROBIAL PATHOGENS

**Fig. (1):**
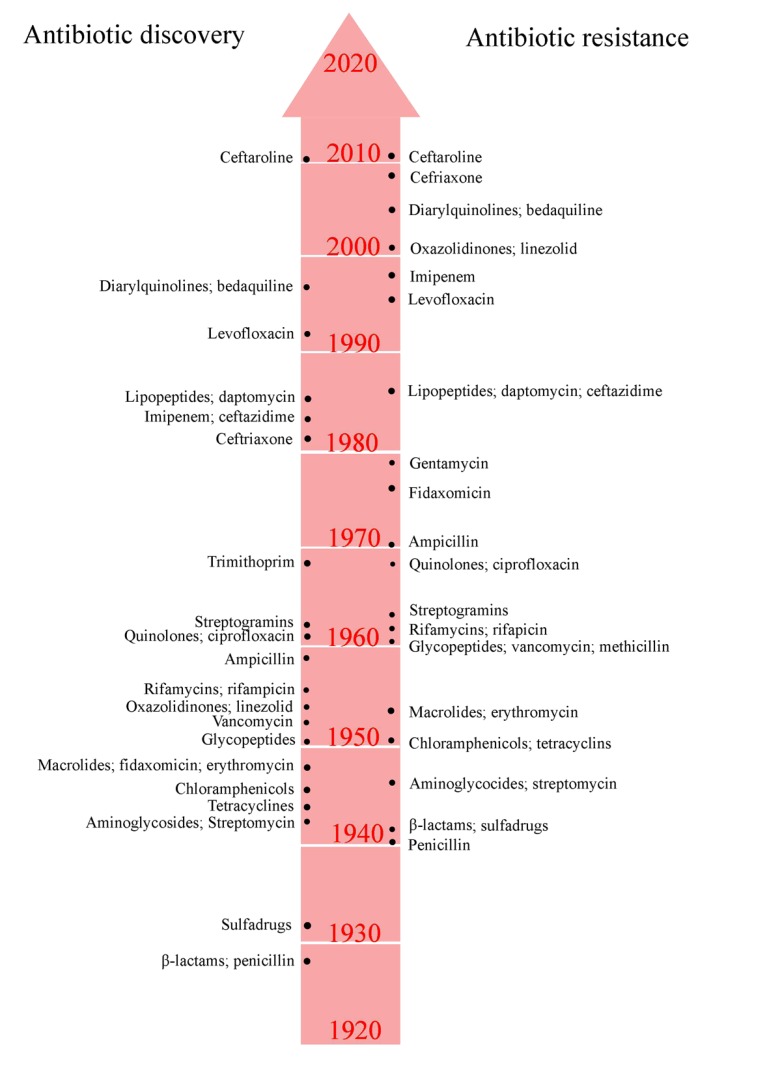
Descriptive timeline of discovery and resistance of antibiotics.

Various antimicrobial agents, effective previously, are no longer useful today because of rise of resistance genes in the microbial genome [20]. Resistance genes emerge through natural selection in the environment over long period of time or by spontaneous mutation in the microbial DNA [21]. Resistant pattern has been reported by almost all antibiotics that have been developed so far (Fig. **1**). The infections caused by antimicrobial-resistant microorganisms often fail to respond to the standard treatment or drug therapy, which result prolonged illness and fatal risk [22].

The main cause of premature mortality and morbidity in Nepal are from bacterial origin. Major infections include acute respiratory infections, diarrheal disease, tuberculosis, and bloodstream infections. For inpatient morbidity, out of 287,616 hospitalized patients in 2014-2015, 11,529 patients were hospitalized due to diarrhea and gastroenteritis followed by other chronic obstructive pulmonary disease (8,053) and unspecified acute lower respiratory infections (7,881), which were the leading cause for hospitalization [23]. Pneumonia, diarrhea, and sepsis are the major health risk for neonates and infants. However, under-five, the infant and neonatal mortality in Nepal have been decreased by 79.59% in the year 1990-2015 [24]. There were 502 new diarrheal cases per 1,000 children under five years in 2014-2015 and number of diarrheal death were 80 [24].

### 2.1 Enteric Pathogens

Enteric microbial pathogens are those that cause severe diarrhea and dysentery which include rotavirus, *Shigella* spp., *Vibrio cholerae*, *Salmonella* spp., enterotoxigenic *Escherichia coli* (ETEC), enteroaggregative *Escherichia coli* (EAEC) and *Campylobacter* spp [25]. In most of the diarrheal cases, antibiotics are not required for complete recovery except some complications like bloody diarrhea. However, antibiotics are often used to treat in most diarrheal cases inappropriately [26].

*Vibrio cholerae* is a causative agent for severe watery diarrhea, which can lead to dehydration and even death. It is usually caused due to contaminated water or food. In Nepal, cholera outbreak is still a serious issue. Nearly, all *Vibrio cholerae* isolates (clinical and environmental) were resistant to cotrimoxazole, nalidixic acid, furazolidone, erythromycin, and ampicillin [27-30]. In addition, resistant strains of *Vibrio cholerae* were also reported for antibiotics chloramphenicol and ciprofloxacin (Table **1**).

In the study of *Salmonella* and *Shigella* spp., most of the species were reported to have multi-drug resistance [31-35]. Cotrimoxazole and nalidixic acid were found to be 100% resistant towards 15 isolates of *Shigella boydii* and ampicillin was unable to inhibit 6 isolates of *Shigella sonnei* [31]. Multi-drug resistant species of *Salmonella* and *Shigella* were well distributed, which have attributed Shigellosis and Salmonellosis to the public health. A systematic meta-analysis of antibiotic resistance conduced for 2 decades (1993-2011) showed that two species of *Salmonella* (*Salmonella* Typhi and *Salmonella* Paratyphi A) were responsible for typhoid and paratyhoid enteric fever [36]. For both strains, *Salmonella* Typhi and *Salmonella* Paratyphi A, resistance to nalidixic acid and ciprofloxacine were sharply increased. However, for both strains, resistance to first-line antibiotics chloramphenicol and cotrimoxazole were in decreasing trends [36]. In contrast, nalidixic acid was more resistant compared to chloramphenicol and cotrimoxazole. These results suggest that the chloramphenicol and cotrimoxazole are still useful for typhoid and paratyhoid enteric fever treatment (Table **2**).

### 2.2 Uropathogens

Urinary Tract Infection (UTI) is one of the most common infectious diseases caused by *E. coli*. In addition, *Klebsiella* spp., *Enterococcus* spp., *Enterobacter* spp., *Citrobacter* spp., and *Proteus* spp. are also associated with UTI. A report by Nepal’s National Public Health Laboratory demonstrated that the resistance rates of *E. coli* for various antibiotics amoxyicillin, cefixime, nalidixic acid, ceftazidime, ciprofloxacin, cotrimoxazole, norfloxacin, ofloxacin, and cefotaxime were above 50% and showed increased trend of antibiotic resistance in the year 2006 to 2010 [37]. Extended Spectrum Beta Lactamase (ESBL) producing *E. coli* exhibited 100% resistance to cephalosporins which revealed ineffectiveness in the treatment of UTI (Table **3**). However, MDR *E. coli* and ESBL *E. coli* were susceptible (100%) to tigecycline, colistin, and amikacin reserving antimicrobial treatment [38, 39].

### 2.3 Pneumococcal Pathogens

Pneumococcal disease is an inflammatory condition of the lung. *Streptococcus pneumoniae*, *Klebsiella pneumoniae*, *Staphylococus aureus*, *Haemophilus influenza* type b (Hib), and *Pseudomonas aeruginosa* are common bacteria that are responsible for pneumonia in Nepal [26]. Common antibiotics used for pneumonia treatment in Nepal were cotrimoxazole, amoxicillin, and chloramphenicol [40]. In contrast, antimicrobial resistance to commonly used antibiotics ciprofloxacin and cotrimoxazole were highly increased from 2000 to 2008 [41]. Various studies reported that most of the antibiotics resistant strains of *Streptococcus pneumoniae* and *Klebsiella pneumoniae* were from clinical isolates of respiratory infections [42-46]. The antibiotics resistant for *Klebsiella* spp., *Streptococcus pneumoniae*, *Haemophilus influenzae*, and *Pseudomonas aeruginosa* are constantly increasing in recent years (Table **4**) [47-49].

### 2.4 Bacteremic Pathogens

Bacteremia is well known as bacterial bloodstream infections. Serious bacterial infections include neonatal sepsis, meningitis, cellulitis, osteomyelitis, brain abscesses, pneumonia, and typhoid [50]. These infections are often serious and possibly resulting in death which requires prompt antibiotic treatment. Out of 120 isolates, 30.8% neonatal sepsis positive cases were observed in neonatal intensive care unit of Nepal Medical College Teaching Hospital (NMCTH), Kathmandu, Nepal. Among them, 56.8% were resulted from *Staphylococcus aureus* infection followed by *Klebsiella pneumoniae* (21.7%), *Pseudomonas aeruginosa* (13.4%) and others [51]. However, the resistance over different antibiotics was also frequent. Studies of sepsis infections in different hospitals reported the resistance of *Staphylococcus aureus*, *Klebsiella pneumoniae*, *Pseudomonas* spp., *Acinetobacter* spp., *Enterobacter* spp., *Citrobacter* spp., *E. coli*, and *Proteus mirabilis* ranged from 25 to 100% against commonly used antibiotics oxacillin, erythromycin, clindamycin, penicillin, cephalexin, cotrimoxazole, gentamicin, amikacin, ofloxacin, cefixime, cefotaxime, ceftazidime, piperacillin, imipenem, piperacillin-tazobactam, and ampicillin [51-57].

### 2.5 Tuberculosis Pathogens

Tuberculosis (TB) is an infectious disease caused by *Mycobacterium tuberculosis*. Resistance of *M. tuberculosis* to first line drugs isoniazid and rifampicin were extensively being increased [58]. The results of drug resistance survey (2011-2012) showed that the levels of drug resistance were high in Nepal, with nearly 9.3% of new patients and resistance among treatment cases were 15.4% [59]. In addition, the trends of Multi-Drug Resistant Tuberculosis (MDR-TB) were increased from 18.6% to 22.3% in the years 2010–2014 [59]. Furthermore, 61 new MDR-TB cases were registered in 2014 to 2015 [60]. These studies showed that the prevalence of resistance to the first-line tuberculosis drugs rifampicin and isoniazid against MDR-TB has been increased in Nepal.

### 2.6 Nosocomial Pathogens

Nosocomial infection is a major Healthcare Associated Infection (HCAI) in Nepal. HCAI and antimicrobial resistance were the principal threats to the patients of intensive care unit [61]. High prevalence of Methicillin-resistance *Staphylococcus aureus* (MRSA) and other bacteria were reported in most of the HCAI studies [62-69]. Currently, in Nepal, MDR *S. aureus* and MRSA is a major clinical threat to public health. One of the major consequences of reporting high rates of multi-drug resistant MRSA is exploitation of vancomycin (Table **5**).

### 2.7 Sexually Transmitted Pathogens

Syphilis and gonorrhea are sexually transmitted infections of mucous membrane surfaces caused by *Treponema pallidum* and *Neisseria gonorrhoeae*, respectively. Studies on antibiotic resistance against sexually transmitted infections remain limited in Nepal. However, few identified studies reported high rate of resistance of *Neisseria gonorrhoeae* to antibiotics penicillin, tetracycline, and ciprofloxacin [70-72].

### 2.8 Wound-Infection Pathogens

Wound-infection is one of the crucial health problem caused by the invasion of pathogenic microbes. Wound is an injury to the body by laceration or breaking of skin either from surgery, accident, war, animal bites or violence [73]. Post-operative wound-infections and injuries among children are the major health risks in Nepal [74-77]. Both gram positive and gram negative bacteria are associated with wound-infection. Most of the identified studies have reported *S. aureus*, *S. epidermidis*, MRSA, *E. coli*, *K. pneumoniae*, *P. aeruginosa*, *Proteus vulgaris*, *Proteus mirabilis*, *Enterococcus* spp., *Enterobacter* spp., and *Acinetobacter* spp. were associated with wound-infections [74-79]. Common antibiotics used for wound infections were amoxicillin (41-70% resistant), amikacin (16-80% resistant), gentamicin (19-75% resistant), cotrimoxazole (37-100% resistant), ofloxacin (23-100% resistant), ciprofloxacin (20-100% resistant), and cephalexin (40-100% resistant) [76-79]. The increasing multi-drug resistant wound infections are the serious issue. *S. aureus* and *E. coli* remained the most frequently isolated etiological agent for wound infection [74, 75, 78, 79]. In addition, hospital acquired wound infection; especially post operational infection has severe consequences on health and wealth burden for In-patients.

## 3 PREVENTIVE MEASURES

The antimicrobial resistance is a huge prime global hurdle and exponentially increasing in Nepal as well and must be addressed promptly and appropriately. Prescribing antimicrobial drugs unnecessarily, over and under dose medication of antibiotics, and unauthorized antibiotic dispensing by drug retailers are principal issues for rapid growth of antimicrobial resistance [13, 14, 16, 17]. Increasing antimicrobial resistance prolongs the illness and results failure with first-line antimicrobial drug treatment which may urge to treat with second-line or third-line drugs [14]. This is usually more expensive than first-line drugs and leads financial burden to the healthcare authorities.

Overall, antimicrobial resistance is increasing enormously. To cope with this problem discovery of new antibiotics may be choice of alternatives. But, only few novel antibiotics are being discovered in past several years. This may create a serious threat in upcoming days to the world’s public health. Furthermore, medical cost due to antimicrobial resistance is also increasing in similar pattern. Here, we recommend some strategies to reduce antimicrobial resistance and to improve effectiveness of antibiotics in the context of Nepal based on World Health Organization (WHO) policy package to combat the spread of antimicrobial resistance on World Health Day, 2011 [80].

- Adopt the guidelines of proper antibiotic use in the hospitals and community healthcare centers.
- Improve the public health issues and find the path to reduce the need for antibiotics (Proper immunization may be a choice to reduce the use of antibiotics).
- Increase surveillance and antibiotic tracking system.
- Make strong policy for antibiotic dispensing by drug retailers.
- Ensure medical personnel to prescribe only essential drugs of assured quality (even medical personnel prescribe more than one antibiotics for a common disease).
- Regulate and promote rational use of medicines.
- Reduce the use of antimicrobial agents in agriculture and animals.
- Raise the awareness programs about antibiotic resistance and public health crisis.
- Educate the public, policy makers, and health professionals on sustainable use of antibiotics.
- Nosocomial infection should be controlled to minimize the spread of resistant bacteria.
- Prevent transmission of bacterial infections.

## CONCLUDING REMARKS

Various species of gram positive and gram negative bacteria are responsible for bacterial infections to humans and animals. Majority of the bacterial isolates are resistant to commonly used antibiotics. Antimicrobial resistance is a consequential concern for Nepal as well as for all countries in the world. Over use, under use, and misuse of antibiotic is a leading cause for its resistance. The lack of proper antibiotic tracking system, AMR (antimicrobial resistance) surveillance, and facilitated laboratories are principal difficulties of Nepal. The appropriate use of antimicrobial drugs and control of spreading resistant bacteria help to maintain the effectiveness of antibiotics. A continuous monitoring and studies on the multidrug resistant bacterial isolates are important measures. In addition, national strategic approach to use antibiotics is utmost emergence to preserve effectiveness of antibiotics for future.

## Figures and Tables

**Table 1:** Antibiotic resistance in *Vibrio cholerae*.

Microorganism	Study Area	No. of Isolates	Antibiotics	Resistance (%)	Reference
*Vibrio cholarae* (Clinical isolate)	Kathmandu city	22	Ampicillin	100	[27]
			Nalidixic acid	100	
			Cotrimoxazole	100	
			Erythromycin	90.9	
			Cefotaxime	18.2	
			Chloramphenicol	9.1	
			Ciprofloxacin	9.1	
*Vibrio cholarae* (Environmental isolate)	Kathmandu city	2	Ampicillin	100	[27]
			Nalidixic acid	100	
			Cotrimoxazole	100	
			Erythromycin	100	
			Chloramphenicol	50	
*Vibrio cholarae*	National Public Health Laboratory, Kathmandu	31	Ampicillin	100	[28]
			Cotrimoxazole	100	
			Ciprofloxacin	6.45	
			Chloramphenicol	3.23	
*Vibrio cholarae*	National Public Health Laboratory, Kathmandu	57	Nalidixic acid	100	[30]
			Cotrimoxazole	100	
			Furazolidone	100	
			Erythromycin	32	
			Ampicillin	26	

**Table 2:** Antibiotic resistance in *Salmonella* spp. and *Shigella* spp.

Microorganism	Study Area	No. of Isolates	Antibiotics	Resistance (%)	Reference
*Shigella flexneri*	Nepalgunj Medical College and Teaching Hospital	29	Ampicillin	96.55	[31]
			Nalidixic acid	96.55	
			Cotrimoxazole	72.41	
			Ciprofloxacin	62.07	
			Ceftazidime	44.83	
			Ofloxacin	37.93	
			Ceftriaxone	34.48	
*Shigella dysemteriae*		19	Nalidixic acid	94.74	
			Cotrimoxazole	84.21	
			Ampicillin	73.68	
			Ciprofloxacin	68.42	
			Gentamicin	36.84	
			Ofloxacin	21.05	
*Shigella boydii*		15	Cotrimoxazole	100	
			Nalidixic acid	100	
			Ampicillin	73.33	
			Gentamicin	33.33	
			Cefotaxime	26.67	
*Shigella sonnei*		6	Ampicillin	100	
			Nalidixic acid	83.33	
			Cotrimoxazole	83.33	
			Ciprofloxacin	33.33	
*Shigella* spp.	National Public Health laboratory, Kathmandu	21	Ampicillin	71.42	[32]
			Cotrimoxazole	66.66	
			Mecillinam	61.9	
			Nalidixic acid	47.62	
			Ciprofloxacin	23.8	
*Salmonella* spp.		9	Nalidixic acid	44.44	
			Ampicillin	33.33	
			Chloramphenicol	33.33	
			Cotrimoxazole	33.33	
*Shigella flexneri*	Tribhuvan University Teaching Hospital (TUTH), Kathmandu	12	Amoxycillin	83.33	[33]
			Ampicillin	66.66	
			Tetracycline	66.66	
			Cotrimoxazole	58.33	
			Ciprofloxacin	58.33	
			Azithromycin	33.33	
			Ceftazidime	8.33	
*Shigella sonnei*		3	Nalidixic acid	100	
			Cotrimoxazole	100	
			Ciprofloxacin	100	
			Tetracycline	33.33	
*Salmonella Typhi*	Alka Hospital, Jawalakhel	56	Nalidixic acid	91.1	[34]
			Ampicillin	1.8	
*Salmonella* Paratyphi A		30	Nalidixic acid	90	
			Chloramphenicol	3.3	
			Ciprofloxacin	3.3	
*Salmonella* spp.	Kathmandu Model Hospital, Kathmandu	83	Nalidixic acid	83.1	[35]
			Ciprofloxacin	3.6	
			Ampicillin	2.4	
			Cotrimoxazole	1.2	
			Chloramphenicol	1.2	

**Table 3:** Antibiotic resistance in *Escherichia coli*.

Microorganism	Study Area or Hospital	No. of Isolates	Antibiotics	Resistance (%)	Reference
*E. coli* (ESBL)*	National Kidney Center, Vanasthali, Kathmandu	18	Cefotaxime	100	[38]
			Ceftazidime	100	
			Ceftriaxone	100	
			Cefixime	94.44	
			Cefalexin	94.44	
			Nalidixic acid	94.44	
			Norfloxacin	94.44	
			Ofloxacin	88.89	
			Ciprofloxacin	88.89	
			Doxycycline	72.22	
			Cotrimoxazole	61.11	
			Nitrofurantoin	27.78	
			Amikacin	0	
*E. coli* (ESBL)	Manamohan Medical College and Teaching Hospital	288	Ampicillin	100	[39]
			Amoxicillin	100	
			Cefixime	100	
			Ceftazidime	100	
			Ceftriaxone	100	
			Aztreonam	100	
			Cephalexin	92	
			Ciprofloxacin	78	
			Tigecycline	0	
			Colistin	0	
*E. coli* (MDR)		480	Ampicillin	100	
			Amoxicillin	84.7	
			Cephalexin	81.6	
			Ciprofloxacin	80.6	
			Cefixime	65	
			Ceftazidime	64	
			Aztreonam	61	
			Levofloxacin	51	
			Cotrimoxazole	33	
			Tigecycline	0	
			Colistin	0	

* ESBL, extended spectrum beta lactamase.

**Table 4:** Antimicrobial resistant in *Pseudomonas aeruginosa*, *Klebsiella* spp. *Streptococcus pneumoniae*, and *Haemophilus influenzae*.

Microorganism	Study Area	No. of Isolates	Antibiotics	Resistance (%)	Reference
*Pseudomonas aeruginosa*	Tribhuvan University Teaching Hospital (TUTH)	24	Ceftazidine	91.6	[47]
			Ciprofloxacin	95.8	
			Levofloxacine	87.5	
			Imipenem	62.5	
			Gentamycin	62.5	
			Cotrimoxazole	0	
			Tigecycline	0	
		37	Cefotaxime	100	
*Klebsiella* spp.			Cefepime	100	
			Cotrimoxazole	100	
			Ciprofloxacin	86.4	
			Gentamycin	83.7	
			Levofloxacine	72.9	
			Penicillin	3.57	
			Tigecycline	0	
*Streptococcus pneumoniae*	Kanti Children's Hospital, Kathmandu	22	Cotrimoxazole	67.86	[48]
			Erythromycin	7.14	
			Cefotaxime	3.57	
*K. pneumoniae*	Mid and far western region, Nepal	36	Penicillin	88.89	[49]
			Ampicillin	44.44	
			Gentamycin	69.44	
			Ciprofloxacin	22.22	
			Chloramphenicol	47.22	
			Erythromycin	30.56	
			Tetracycline	52.78	
			Cotrimoxazole	52.78	
*S. pneumoniae*		30	Ampicillin	56.67	
			Cotrimoxazole	63.33	
			Penicillin	90	
			Chloramphenicol	40	
			Gentamycin	13.33	
			Erythromycin	33.33	
			Ceftriaxone	0	
*Haemophilus influenzae*		68	Ampicillin	54.41	
			Penicillin	91.18	
			Cotrimoxazole	47.06	
			Chloramphenicol	32.35	
			Gentamycin	16.18	
			Tetracycline	41.18	
			Ciprofloxacin	16.18	

**Table 5:** Antibiotic resistance in *Staphylococcus aureus* and Mehicillin-resistant *Staphylococcus aureus* (MRSA).

Microorganism	Study Area or Hospital	No. of Isolates	Antibiotics	Resistance (%)	Reference
*S. aureus*	Chitwan Medical College Teaching Hospital, Chitwan	306	Penicillin	94.7	[62]
			Cotrimoxazole	81.7	
			Cephalexin	68	
			Gentamicin	60.4	
			Ciprofloxacin	63.7	
			Erythromycin	32.7	
			Cefoxitin	43.1	
			Oxacillin	39.2	
			Clindamycin	27.5	
			Amikacin	10.7	
			Vancomycin	0	
			Teicoplanin	0	
*S. aureus*	Universal College of Medical Sciences Teaching Hospital, Bhairahawa	162	Penicillin	81.5	[67]
			Erythromycin	71.7	
			Ampicillin	87.4	
			Amoxicillin	91.9	
			Tetracycline	39.6	
			Ciprofloxacin	26.5	
			Amikacin	19	
			Cloxacillin	69.1	
			Vancomycin	0	
MRSA		112	Penicillin	100	
			Cloxacillin	100	
			Amoxicillin	91.8	
			Ampicillin	90	
			Erythromycin	68.7	
			Cephalexin	66.03	
			Cefazolin	57.6	
			Vancomycin	0	
MRSA	Kathmandu Medical college Teaching Hospital, Kathmandu	29	Penicillin	100	[69]
			Oxacillin	100	
			Cephalexin	75.86	
			Cotrimoxazole	44.82	
			Erythromycin	44.82	
			Tetracycline	20.68	
			Gentamicin	20.68	
			Amikacin	24.13	
			Ciprofloxacin	17.03	
			Vancomycin	0	
